# Effectiveness of Physiotherapy Intervention in Guillain Barre Syndrome: A Case Report

**DOI:** 10.7759/cureus.52062

**Published:** 2024-01-10

**Authors:** Ishwari Gawande, Aditi Akhuj, Snehal Samal

**Affiliations:** 1 Department of Neuro-Physiotherapy, Ravi Nair Physiotherapy College, Datta Meghe Institute of Higher Education and Research, Wardha, IND

**Keywords:** functional independence scale, rehabilitation, huges scale, physiotherapy management, guillian-barre syndrome

## Abstract

Guillain-Barre syndrome (GBS) is described by a wide range of motor impairment, flaccidity, hyporeflexia, and progressive and ascending flaccid paralysis. Group B *Streptococcus*, also known as *Streptococcus agalactia* and *Campylobacter jejuni*, are Gram-positive bacteria also known as the leading cause of GBS; its variants are acute motor axonal neuropathy (AMAN), acute motor and sensory axonal neuropathy (AMSAN), acute inflammatory demyelinating neuropathy (AIDP), and Miller-Fisher syndrome (MFS). A 20-year-old girl came with complaints of generalized weakness, fever and pain over the lower limb. Huges (GBS disability scale) and the Functional Independence Measure scale were used for recording the outcome measures, and treatment has been demonstrated to lessen challenges and enhance health and quality of life. The rehabilitation protocol results in the improvement of posture motor control and the avoidance of secondary impairments.

## Introduction

Guillain-Barre syndrome (GBS) is unusual inflammatory demyelinating polyradiculoneuropathy that results from loss of motor control. The most common forms responsible for 70-80% of cases are acute motor axonal neuropathy (AMAN) and acute inflammatory demyelinating neuropathy (AIDP), and others are Miller-Fisher syndrome (MFS) and acute axonal and sensory axonal neuropathy (AMSAN), which are the variants of GBS [1]. Numerous clinical, neurophysiological, and pathological studies have suggested that acute inflammatory neuropathy primarily affects large-diameter myelinated nerves. According to a few articles, experimental allergic neuritis (EAN), which can be caused by immunizing animals, is thought to have pathological alterations in the peripheral nervous system (PNS), which is seen in GBS [2]. Patients with GBS experience sensory abnormalities like sensory ataxia, allodynia, and a reduction in sensation to thermal or nociceptive stimuli [3]. Flaccidity is seen, which initially affects the lower limbs and advances in an ascending manner; in this, the patient experiences weakness in the lower limb with tingling or numbness with the appearance of "rubber legs" or legs that buckle [4]. The primary pathogen is *Campylobacter jejuni*, known to be a leading cause of GBS, which influences the immune response that targets the infecting organisms and interacts with the brain tissue. AIDP is caused by an immune system reaction against Schwann cells present over the surface membrane in 85% and a response against an epitope in the axonal membrane in 15% of cases [5]. Studies have shown patients with good physical fitness suffering from GBS have a good recovery rate in mental functioning and motor control [6]. Pain is seen as prevalent in 66% of cases of acute GBS patients [7].

Physiotherapy treatment aims to accelerate recovery and lessen the severity of the condition by treating symptoms like weakness, pain, and difficulty in breathing through breathing exercises, strengthening, mobility, and postural control. Continuation of therapy will help the patient go back to her daily routine and make her functionally independent [8]. This case presents a 20-year-old with complaints of weakness in the lower extremity that progressed into flaccidity of all four limbs and severe pain in the lower limb; she was not able to perform the coordinated movement, and the management was begun by providing ventilator support and hospitalization the patients, specific immunomodulating pharmaceuticals that seem to minimize nerve damage was used, the physiotherapy rehabilitation was begun from the day of admission.

## Case presentation

Patient information

A 20-year-old girl visited Acharya Vinobha Bhave Rural Hospital (AVBRH) hospital with complaints of bilateral upper limb as well as lower limb weakness; her lower limbs were more affected than her upper limbs, which started before coming to the hospital. She also complained of severe pain in her lower limb with a tingling sensation, motor dysfunction, abnormal gait and severe difficulty in walking. She gave a history of fever 15 days back. The patient had no history of ear, nose, throat bleed, hemoptysis, head trauma, or seizures and gave no relevant medical history of tuberculosis, bronchial asthma, diabetes or hypertension. Cerebrospinal fluid (CSF) investigations showed elevated protein levels (111 mg/dL) and raised white blood cell (WBC) count, confirming that the patient had GBS.

Clinical findings

The patient was conscious and cooperative, with a Glasgow coma scale (GCS) score of E4, V4, and M6. She was afebrile and hemodynamically stable. The patient's oral consent was taken. On inspection, the patient was ectomorphic. On examination, deep and superficial reflexes were diminished, as shown in Table 1. The upper and lower limbs were flaccid (Table 2). Sensations were intact. Pain assessment was done using a visual analogue scale (VAS); the findings were 7/10 at rest and 9/10 on the movement of the lower limb.

**Table 1:** Demonstrates the variation in reflexes grading 0: Absent, 1+: Present but depressed, 2+: Brisk response; normal, 3+: Very response, 4+: Clonus always abnormal

Types of reflex	Pre-intervention
Superficial reflexes	
Planter	1+
Abdominal	1+
Deep tendon reflexes	
Biceps	1+
Triceps	1+
Knee jerk	1+
Ankle jerk	1+

**Table 2:** Demonstrate the variation in muscle tone (TGS) Tone grading system (TGS): 0: No increase in tone, 1+: Decrease tone, 2+: Normal tone, 3+: Increase tone

Muscle group	Pre-intervention
Shoulder flexors	0
Shoulder extensors	0
Elbow flexors	0
Hip extensors	0
Hip flexors	0
Ankle flexors	0
Ankle extensors	0

Diagnostic assessment

Primarily, the weakness was seen in the distal muscle group, which later progressed to proximal muscles. Deep tendon reflexes were absent in the patient; CSF examination was performed as a diagnostic tool, which revealed an increased protein level (111 mg/dL). She was diagnosed with AIDP following the nerve conduction velocity findings, which demonstrated reduced and prolonged distal motor latency, as well as conduction velocity within normal limits in the bilateral median, ulnar, and tibial nerves. Additionally, the amplitude of the compound muscle action potential could not be elicited in the bilateral peroneal nerve. The bilateral ulnar, tibial, peroneal, and median nerves were unable to elicit F-min latency. The amplitude of the sensory nerve action potential was diminished in the bilateral median nerves and was not elicitable in the right ulnar or bilateral sural nerves.

Therapeutic intervention

After admission to the hospital, early rehabilitation was initiated. The treatment aimed to maximize neuromuscular function and to gain back patient's functional independence, thereby improving quality of life. Intravenous immunoglobulin (IVIG) was given instead of plasma exchange as the primary treatment, with a dose of 0.4/kg body weight daily for five days regularly. Relaxation was the basic pain management given. Table 3 gives a brief summary of the physiotherapy intervention received by the patient. 

**Table 3:** Physiotherapy management PROM: Passive range of motion, TA: Tendo Achilles, PNF: Proprioceptive neuromuscular facilitation, Reps: Repetitions, UL: Upper limb, LL: Lower limb, Sec: Seconds, TENS: Transcutaneous electrical nerve stimulation

Goals	Treatment protocol	Doses
To reduce flaccidity	PROM to UL and LL was given to the patient to mobilize the joints and minimize stiffness.	20 reps with a break of 2 minutes.
To prevent contractures	Passive contract-relax stretches were given to TA.	2 sets of 10 reps each with 30 sec of hold and 5 sec of release.
To normalize the muscle tone	PNF techniques for both UL and LL were given.	Contract relax technique is given for 10 reps in 1 set.
To improve muscle strength	A combination of isometric and rhythmic stabilization was given to LL, dynamic quadriceps.	10 reps in 1 set.
To alleviate tingling sensation	TENS	10 mins, 2 times per day.
To prevent respiratory complications	Segmental breathing and pursed lip breathing exercises.	10 reps of each exercise.
To enhance functional status and bed mobility	Supine-to-sit transition training with rolling facilitation.	Employing upper extremity momentum and crossing the ankles to roll from a supine position to a side-lying position, transition training from supine to sitting with prone on the elbows from prone on the elbow to long sitting, use elbow walking, supine on the elbow.

**Figure 1:**
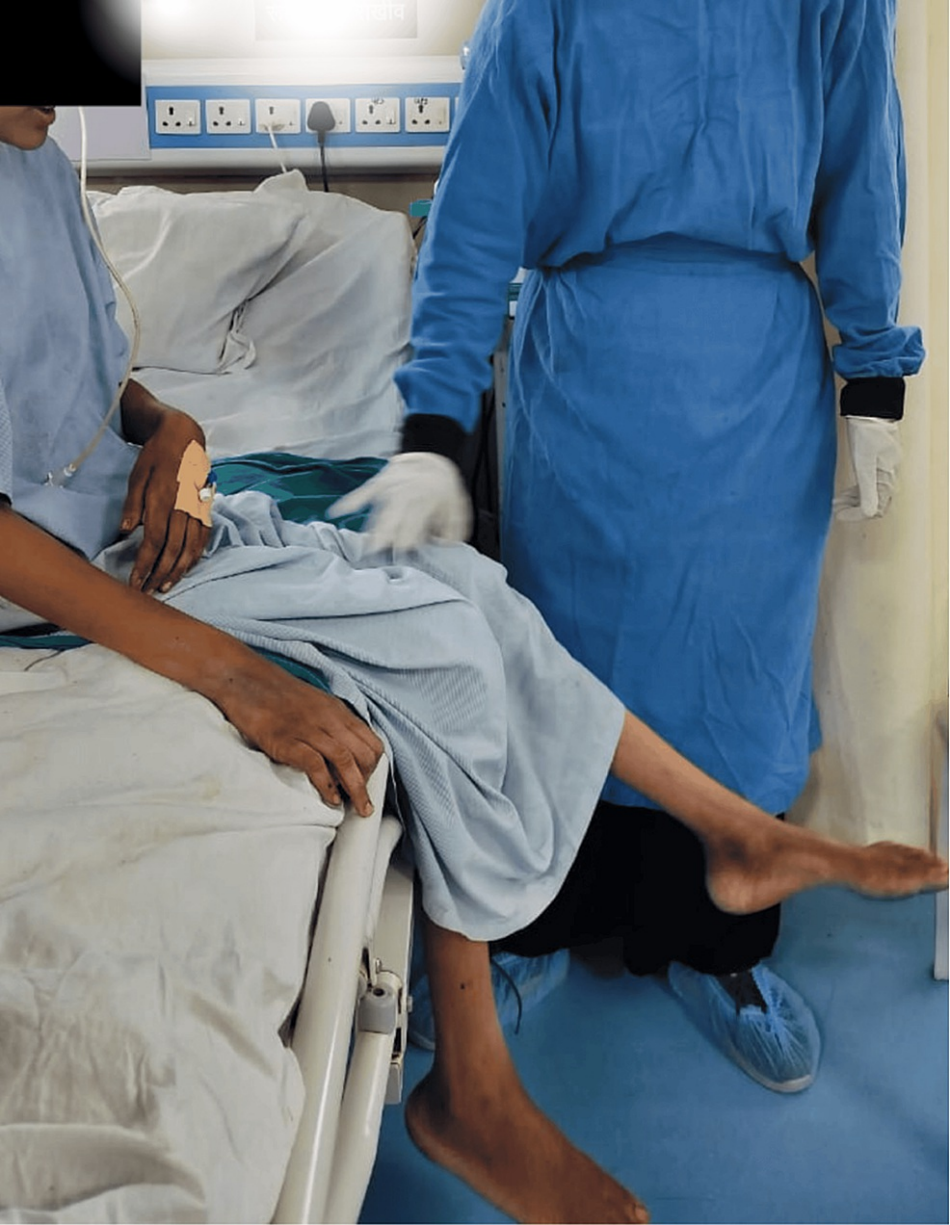
Dynamic Quadriceps

 Figure 1 shows the patient performing dynamic quadriceps.

Follow-up and outcome measures

The ultimate result of neurorehabilitation was to see improvement in a 20-year-old patient suffering from GBS at the end of the session, which is summarized in Table 4-6 below. The outcome measures used are summarized in Table 7. 

**Table 4:** Demonstrates the variation in reflexes grading. 0: Absent, 1+: Present but depressed, 2+: Brisk response; normal, 3+: Very response, 4+: Clonus always abnormal.

Types of reflex	Post-intervention (Day 30)
Superficial reflex	
Planter	2+
Abdominal	2+
Deep tendon reflexes	
Biceps	2+
Triceps	2+
Knee	2+
Ankle	2+

**Table 5:** Demonstrates the variation in muscle tone (TGS) Tone grading system (TGS): 0: No increase in tone, 1+: Decrease tone, 2+: Normal tone, 3+: Increase tone

Muscle group	Post-intervention (Day 30)
Shoulder flexors	2 +
Shoulder extensors	2 +
Elbow flexors	2 +
Hip extensors	2 +
Hip flexors	2 +
Ankle flexors	2 +
Ankle extensors	2 +

**Table 6:** Demonstrates the grades of manual muscle testing (MMT) 0: No contraction with flickering contraction, 2: Full range of motion (ROM) with gravity elimination, 3: Full ROM against gravity, 4: Full ROM against gravity, moderate resistance, 5: Full ROM against gravity, maximum resistance

Extremity	Pre-intervention (Day 1)	Post-intervention (Day 30)
Upper Extremity	1/5	4/5
Lower Extremity	1/5	4/5

**Table 7:** Outcome measures Huges scale grading: 0: Normal, 1: Slight clinical symptom, 2: Able to walk 5m or more without assistance but unable to run, 3: Able to walk 5m with help, 4: Bedridden or chair bound, 5: Ventilator assisted breathing, 6: Death. NPRS: Numerical pain rating scale

Outcome measures	Pre-intervention (Day 1)	Post-intervention (Day 30)
Huges scale	4	2
NPRS scale	8	5
Functional independence scale	65/120	105/120

## Discussion

GBS is an auto-immune disorder that affects nerves and how they function in the body; however, this case study intended to show how physical rehabilitation has been beneficial for patients suffering from GBS. In this case, improvement is seen as the primary outcome, which also provides additional proof of the patient's recovery with IVIG treatment [9,10] and prolonged exercise [11]. Physical therapy, like massage and relaxation, helped a lot with pain management [12]. IVIG was preferred over the plasma exchange therapy [13,14].

Moreover, proprioceptive neuromuscular facilitation (PNF) techniques have shown quick improvement and have increased neuromuscular efficiency [15,16]. Chest physiotherapy and frequent position changing Franklin's exercises were performed to increase the efficiency of movement, with less risk of injury [17]. Khan et al. carried out a study on outcome measures of low-intensity rehabilitation programs in the acute phase, which showed good results and reduced the disability in patients suffering from GBS in later stages. This also showed a good result in this case. The investigations showed a high protein level and normal white blood cell count, which was done on cerebrospinal fluid, conforms to the diagnosis of GBS [18,19]. Moreover, the patient, after the pro-long duration of treatment, can perform mild exercises that do not require a large amount of strength or endurance [20]. The patient's quality of life and functional outcome were elevated, showing the positive result of physiotherapy treatment. Thus, this case study illustrates how physiotherapy significantly enhances strength, daily living activities and quality of life.

## Conclusions

In conclusion, the physiotherapy intervention in this GBS case report demonstrated notable effectiveness. The patient exhibited improvement in motor function, increased strength, respiratory function and enhanced overall mobility. This case report suggests that early and targeted physiotherapy intervention can contribute significantly to the recovery and quality of life for patients. These positive outcomes underscore the valuable role of physiotherapy in managing Guillain-Barre syndrome, emphasizing its significance in the holistic care and rehabilitation of affected individuals. 
